# Persistent Thrombocytopenia of an Unexplained Cause in a Patient With Metastatic Renal Cell Carcinoma: A Case Report

**DOI:** 10.7759/cureus.76229

**Published:** 2024-12-22

**Authors:** Nidhi Shree, Somnath Dey, Kunal R Vinayak, Pallavi Singh, Durgatosh Pandey

**Affiliations:** 1 Community Medicine, Dr. D. Y. Patil Medical College, Hospital and Research Centre, Dr. D. Y. Patil Vidyapeeth, Pune, IND; 2 Palliative Care, Mahamana Pandit Madan Mohan Malaviya Cancer Centre, Homi Bhabha Cancer Hospital, Tata Memorial Centre, Varanasi, IND; 3 Surgical Oncology, Mahamana Pandit Madan Mohan Malaviya Cancer Centre, Homi Bhabha Cancer Hospital, Tata Memorial Centre, Varanasi, IND

**Keywords:** lymphorrhea, magnesium sulphate, occupation therapy, palliative care, thrombocytopenia

## Abstract

Thrombocytopenia is a common complication in patients with solid tumors, particularly renal cell carcinoma (RCC), arising from mechanisms such as chemotherapy, direct tumor invasion, and paraneoplastic syndromes. Managing thrombocytopenia in advanced cancer presents significant challenges, often limiting therapeutic options and impacting patient outcomes. This case report describes a 62-year-old man with metastatic RCC complicated by persistent thrombocytopenia, unresponsive to both conventional treatments and novel therapies. Initially treated with palliative intent using Sunitinib, the patient developed complex symptoms including breathlessness, lymphorrhea, petechial rashes, and generalized weakness. Symptom management in a palliative care setting focused on pain relief with morphine and fentanyl, pleural effusion drainage, magnesium sulfate application for lymphorrhea, and occupational therapy for functional support. Additionally, psychological and spiritual care provided holistic support to the patient and his family. This case underscores the challenges of managing refractory thrombocytopenia in metastatic RCC and highlights the importance of individualized care in palliative settings. While therapeutic interventions were limited by the complexity of the disease, improvements in symptom control and emotional well-being demonstrate the value of a multidisciplinary approach in enhancing the patient’s quality of life. This report emphasizes the need for further research into effective strategies for managing malignancy-associated thrombocytopenia.

## Introduction

Thrombocytopenia is defined as a platelet count below the lower limit of normal, i.e., less than 150 × 10³ per μL (150 × 10⁹ per L) [1]. In mild cases, the platelet count ranges from 70 to 150 × 10³ per μL (70 to 150 × 10⁹ per L), while in severe cases, it is less than 20 × 10³ per μL (20 × 10⁹ per L) [1].

Patients with a platelet count between mild and severe thrombocytopenia may present with purpura and experience excessive bleeding following trauma. In severe thrombocytopenia, patients present with bleeding even with minimal trauma, whereas counts less than 10 × 10^3^ per μL increase the risk of spontaneous bleeding, petechiae, and bruising. Spontaneous bleeding (i.e., mucosal, intracranial, gastrointestinal, and genitourinary bleeding) is more likely in patients with platelet counts less than 5 × 10^3^ per μL (5 × 10^9^ per L) and is considered a hematologic emergency [2].

Paraneoplastic syndromes associated with malignancy also present as immune-mediated hematological syndromes. These include autoimmune hemolytic anemia (AIHA), increased formation of anti-Factor VIII, and antiphospholipid antibody (APLA), along with immune thrombocytopenic purpura (ITP) and many others like coagulopathy, infection (e.g., cytomegalovirus), drug reaction (e.g., antibiotics like vancomycin and linezolid and antiviral agents like ganciclovir commonly induce thrombocytopenia by direct bone marrow toxicity or drug-dependent antibody clearance), post-transfusion purpura, and thrombotic microangiopathy [1-3]. In adult solid tumors, thrombocytopenia occurs via several mechanisms. Thrombocytopenia is one of the common problems in adult solid tumors after hematological malignancies. It is found that approximately 10% to 38% of patients suffering from solid tumors and 40% to 68% of patients suffering from any hematologic malignancy experience thrombocytopenia [4-8]. The causes are often multifactorial, with the most common being systemic chemotherapy or radiation treatment, which can induce bone marrow hypoplasia [9]. Other causes include direct tumor infiltration into the bone marrow or conditions such as disseminated intravascular coagulation (DIC) and thrombotic thrombocytopenic purpura [3].

The occurrence of thrombocytopenia along with solid tumors is a poor prognostic factor [10]. It adds to further complications in the management of malignancy in these patients leading to their early death. Among all solid tumors, the highest prevalence of thrombocytopenia has been observed in patients with colorectal cancer, followed by non‐small cell lung cancer, ovarian cancer, renal cell cancer (RCC), and others [4].

In RCC patients, thrombocytopenia is mainly attributed to paraneoplastic syndromes, such as secondary ITP (approximately 20% of all RCC patients) [10], followed by other causes, including IL-6-related thrombocytopenia, secondary DIC, and a few unknown causes.

In this report, we describe the presentation and management of a 62-year-old man with metastatic RCC and an unknown cause of secondary thrombocytopenia in a palliative care setting.

## Case presentation

**Figure 1:**
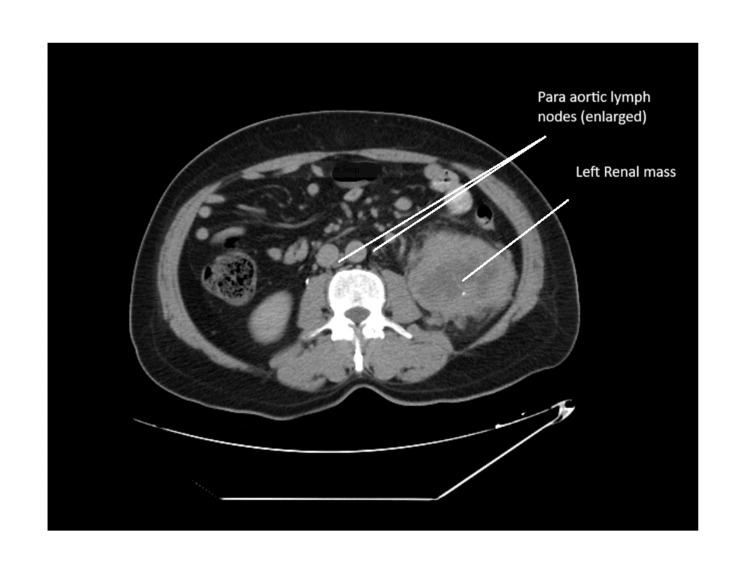
A CT scan of the abdomen A CT scan of the abdomen revealed a left renal mass measuring 10.1 x 7.9 x 13.7 cm, accompanied by enlarged para-aortic lymph nodes measuring 2.4 x 1.8 cm. CT, computed tomography

**Figure 2:**
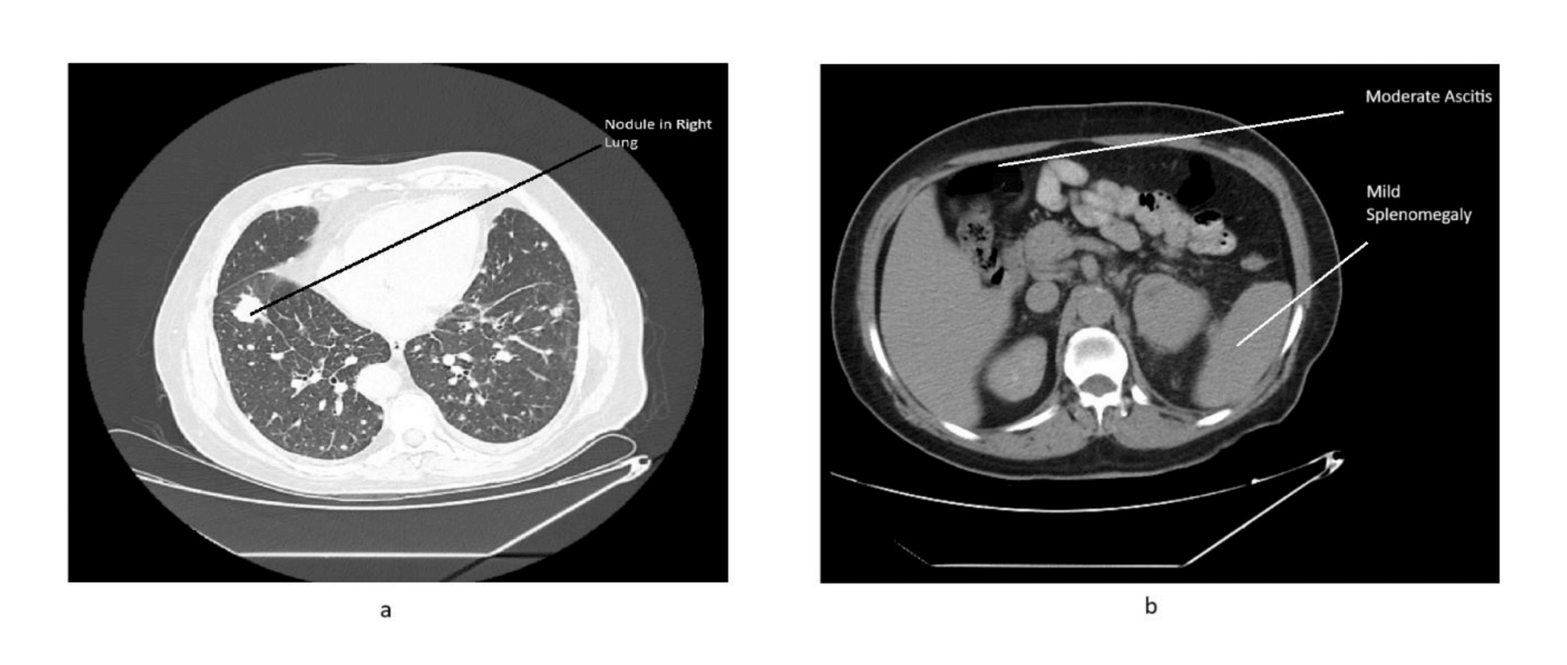
a) CT thorax and b) CT abdomen A CT scan of the abdomen revealed moderate ascites and mild splenomegaly with the spleen measuring 12.4 cm. A CT thorax revealed bilateral lung nodules, with the most prominent visible white rounded lesion in the right lung. CT, computed tomography

A 62-year-old retired government employee from North India, with a history of hypothyroidism for 15 years, hypertension for 10 years, and Hodgkin's lymphoma treated 23 years earlier, presented to the Medical Oncology Department with complaints of chronic cough and abdominal pain for three months, along with generalized weakness for one month. He had no history of substance use. A computed tomography (CT) scan of his abdomen revealed a left renal mass measuring 10.1 × 7.9 × 13.7 cm, along with enlarged para-aortic lymph nodes measuring 2.4 × 1.8 cm, bilateral lung nodules, and other features suggestive of lymphangitis carcinomatosis. Additional findings included bilateral pleural effusion, moderate ascites, and mild splenomegaly with the spleen measuring 12.4 cm (Figures 1, 2).

A CT-guided biopsy, previously performed at another center, was reviewed at the hospital and confirmed the diagnosis of clear cell renal cell carcinoma (RCC). His International Metastatic Renal Cell Carcinoma Database Consortium (IMDC) score was three out of five, indicating a poor prognosis [11].

The patient had a significant medical history, including Hodgkin's lymphoma treated 23 years earlier, with no evidence of recurrence on follow-up. His regular medications included a tablet of amlodipine 10 mg daily for hypertension and a tablet of eltroxin 50 mcg daily for hypothyroidism. After his thyroid function test revealed a thyroid-stimulating hormone (TSH) level of 27.9 mIU/L, his Eltroxin dose was increased to 75 mcg daily. Routine hematological and biochemical workups, including complete blood count (CBC), liver function tests (LFT), renal function tests (RFT), and serum electrolytes, were unremarkable. Echocardiography revealed good cardiac function with a left ventricular ejection fraction of 60%.

Following discussion at a multidisciplinary tumor board, the treatment plan was designed with palliative intent due to the advanced stage of the disease. In view of the patient’s Eastern Cooperative Oncology Group (ECOG) performance score of two, oral sunitinib, a receptor tyrosine kinase inhibitor, was initiated on a two-week-on, one-week-off schedule. After four weeks of therapy, the patient reported improvement in his cough. However, he subsequently developed progressive breathlessness, abdominal distension, and a petechial rash on his abdomen and lower limbs, prompting emergency admission.

On admission, laboratory results showed a hemoglobin level of 9.8 g/dL, white blood cell count of 5.49 × 10⁹/L, absolute neutrophil count of 4.95 × 10⁹/L, and platelet count of 173 × 10⁹/L. The prothrombin time/INR (International Normalized Ratio) was 15.10/1.32. Serum creatinine was mildly elevated at 1.20 mg/dL. His serum albumin was 3.30 g/dL, total bilirubin was 0.35 mg/dL, serum AST (aspartate aminotransferase)/ALT (alanine transaminase) was 23/17 U/L, serum alkaline phosphatase was 184 U/L, serum sodium was 117.5 mmol/L, serum potassium was 4.31 mmol/L, and serum calcium was 10.7 mg/dL.

Given the suspicion of drug-induced thrombocytopenia, sunitinib was discontinued. The poor prognosis was explained to the patient's son and relatives, after which the patient was referred to the Palliative Medicine Department for further symptom management.

Upon evaluation by the palliative care unit, the patient reported breathlessness, generalized pain, and a petechial rash all over the body, more pronounced over the bilateral thighs and abdomen. He also gave a history of melena and weakness in both lower limbs. His performance status had declined to ECOG 3, with grade 3 stomatitis (CTCAE v4.0 grading scales) noted. The ESAS (Edmonton Symptom Assessment Scale) score for breathlessness was 6/10. He also had moderate ascites, bilateral pedal edema, and lymphedema.

**Figure 3:**
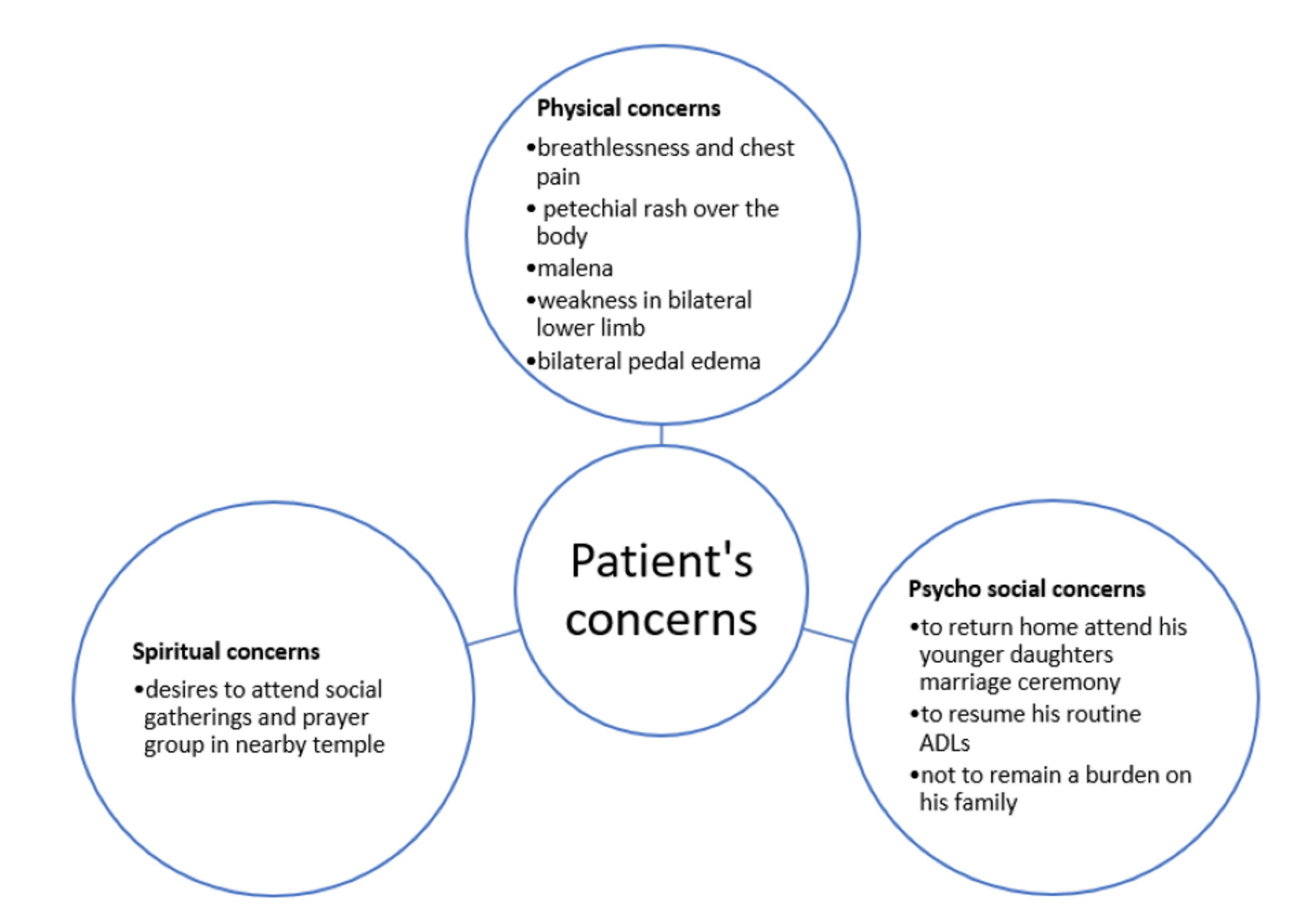
Presentation of patient's concerns This figure describes the physical, spiritual, and psychosocial concerns of the patient.

His psychosocial-spiritual assessment revealed that he was a retired widower, living in a nuclear family, belonging to a middle socioeconomic status as per the Modified Kuppuswamy Socioeconomic Scale [9]. His spiritual inclination was strong in his religious beliefs. The patient was aware of his diagnosis and its prognosis. His son was the primary caregiver and he relied on his son’s decision for the treatment. He had a strong bond and good family support. He found comfort in his strong faith in God and believed that attending social gatherings and prayer services at a nearby temple would help him emotionally. He did not blame himself or God for his current health condition. His main concerns (Figure 3) were to get relief from pain, breathlessness, and other symptoms, as he wanted to return to his routine ADLs (activities of daily living) and not feel like a burden to his family. His primary goal was to manage his symptoms effectively and recover sufficiently to attend his daughter’s wedding, scheduled two months later.

Patient management

Management of Physical Concerns

The patient was initially started on nebulization with Duolin and Budecort to address airway obstruction and inflammation, aiming to improve airflow and relieve breathlessness. However, this provided limited relief. Physiotherapy was also initiated to assist with improving respiratory function and mobility. Subsequently, he was started on a tablet of morphine 2.5 mg every four hours (Q4H) and 2.5 mg PRN (as needed) for relief of breathlessness and pain, along with oral antibiotics to address potential infections. During follow-up visits, his dyspnea gradually improved over the course of a week, but he continued to complain of generalized pain and weakness.

By the second week of his hospital stay, laboratory investigations revealed raised serum creatinine levels of 1.20 mg/dL, indicating mild renal impairment. In response, his pain medication was changed from tablet morphine to a transdermal fentanyl patch (25 mcg/hour, applied every 72 hours) to provide continuous pain relief. Additionally, tablet buprenorphine 0.2 mg was prescribed sublingually (S/L) PRN for breakthrough pain (BTP). To prevent opioid-induced constipation, he was started on a stimulant-lubricant laxative (15 mL HS). For muscle relaxation and neuropathic pain management, tablet Myoril 4 mg BD (twice daily) and capsule gabapentin 100 mg HS (at bedtime) were also added. The injection of paracetamol (PCM) 1 gm IV SOS (as needed) was made available for additional pain relief.

**Figure 4:**
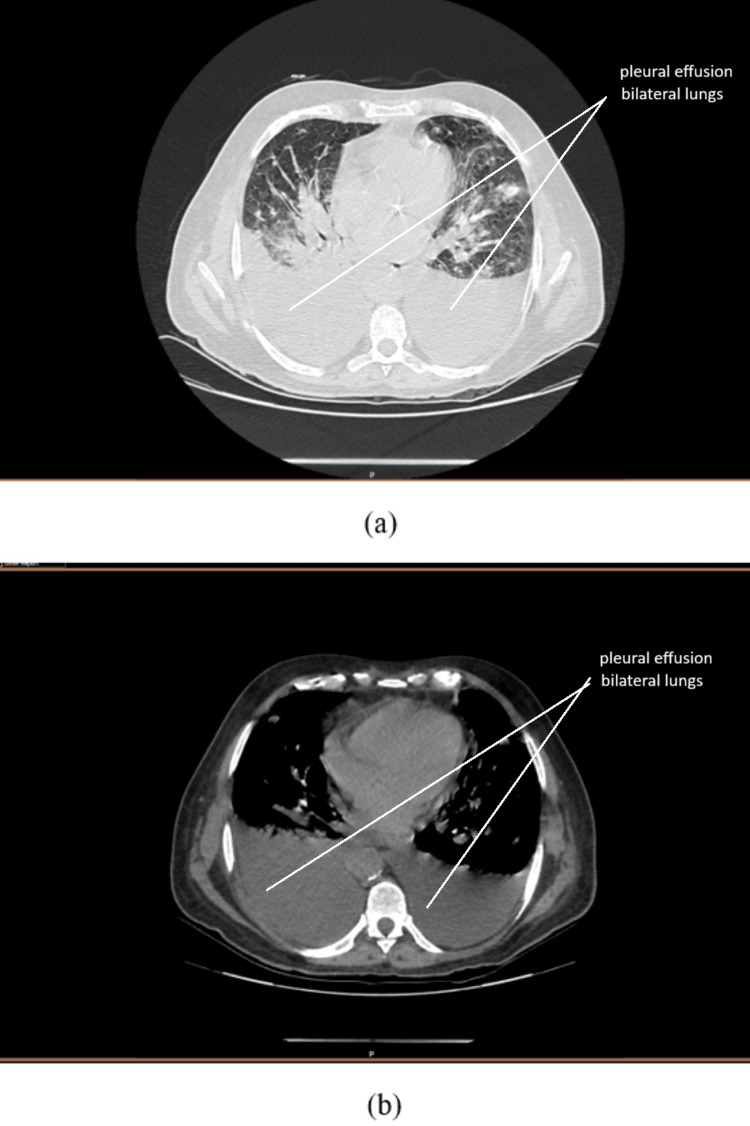
CECT thorax CT revealed a large right-sided effusion and a moderate left-sided effusion, with partial collapse of the bilateral lower lobes. The visualized lung fields showed multiple metastatic nodules of varying sizes. Few patches of consolidation were seen centrally in the lung fields, likely of infective etiology. An enlarged left kidney with perirenal fat stranding and thickened Gerota's fascia, consistent with the known neoplastic status, was also observed. CT, computed tomography; CECT, contrast-enhanced computed tomography

On the following day, the patient reported >95% relief in pain, with no need for any PRN breakthrough doses. The injection of Perinorm (metoclopramide) 10 mg IV TDS (three times daily) was initiated to address nausea and vomiting. Given his persistent breathlessness, a second contrast-enhanced computed tomography (CECT) thorax was advised for further evaluation (Figure 4).

Following the CECT, he underwent right-sided pigtail insertion for pleural effusion, along with regular chest physiotherapy, incentive spirometry, and diaphragmatic breathing exercises. He was also advised to walk for a few meters each day.

Within a few days into the third month of treatment, the patient developed sleep disturbances and delirium. He was initiated on haloperidol 0.25 mg intramuscularly (IM), at bedtime (HS), and administered regularly for eight days to manage these symptoms. For nausea and vomiting, he continued to receive (Perinorm) metoclopramide 10 mg intravenously (IV) SOS. Also, dexamethasone 4 mg IV TDS (at 6 AM, 11 AM, and 4 PM) was administered, followed by a tapering schedule. Additionally, (lasix) furosemide 20 mg IV was given as a stat dose to address fluid overload. Intravenous hydration with normal saline (IV NS) was maintained throughout, but zoledronic acid IV infusion was avoided due to impaired renal function (creatinine clearance: <30 mL/min).

**Figure 5:**
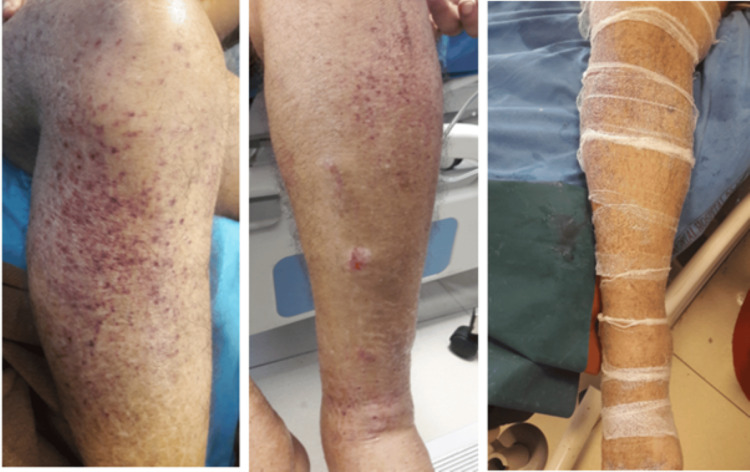
Lymphorrhea in left lower limb with and without magnesium sulfate solution dressing The figure depicts improvement in left lower limb lymphorrhea after the application of magnesium sulfate solution dressing on lymphorrhea in left lower limb.

Later in the third month, the patient developed bilateral lymphedema, likely secondary to hypoproteinemia. He was prescribed intravenous albumin 20% infusion over four hours for three days to correct hypoalbuminemia. The hospital course was further complicated by the onset of visible lymphorrhea from the lower extremities (Figure 5). The occupational therapy department was consulted to initiate magnesium sulfate (topical) dressings, resulting in gradual symptom improvement within three days. In addition, the patient developed hypokalemia (serum potassium: 3.31 mmol/L), for which oral potassium supplements were administered to restore normal electrolyte levels.

During the third month of treatment, the patient developed a worsening petechial rash across his body. Laboratory tests revealed a platelet count of 117 × 10⁹/L, with a downward trend indicating progressive thrombocytopenia, along with hypocalcemia. In response, a single donor platelet (SDP) transfusion (intravenous) was administered, and intravenous calcium gluconate was given to correct the hypocalcemia. A peripheral blood smear showed occasional schistocytes. However, the absence of clinical signs of DIC and a normal fibrinogen level made DIC an unlikely diagnosis.

Given the concern for secondary ITP as a potential paraneoplastic syndrome associated with RCC, dexamethasone 40 mg orally was initiated for four days. Additionally, subcutaneous romiplostim (a thrombopoietin mimetic that stimulates platelet production by megakaryocytes) was administered at a starting dose of 1 μg/kg weekly. Despite these interventions, the patient failed to respond, and follow-up CBC tests revealed a further decline in platelet count to 60 × 10⁹/L after four days of dexamethasone and the first dose of romiplostim.

Severe thrombocytopenia recurred within two days of completing the dexamethasone course. At this stage, oral prednisone 80 mg daily was started, and the romiplostim dose was increased to 3 μg/kg in the second week of the third month. Despite these escalated treatments, the platelet count continued to decline, reaching 33 × 10⁹/L, suggesting that neither ITP as a paraneoplastic syndrome associated with RCC nor sunitinib-induced thrombocytopenia was the underlying cause.

Management of Psychosocial and Spiritual Concerns

Psychological counseling and support were provided to the patient and his caregiver by Medical Social Workers (MSW). The son, serving as the primary caregiver, was also counseled about the intent of treatment and the poor prognosis to help him prepare for future care decisions. The financial aspects of the treatment were managed through family support and coverage under the Central Government Health Scheme (CGHS), easing some of the family’s burden. Despite ongoing clinical challenges, including declining platelet counts and persistent hypocalcemia, the patient’s chief symptoms gradually improved over time, enhancing his overall comfort.

Throughout his illness, the patient remained spiritually connected, finding strength in his religious beliefs and faith in God. His goal was to resume his daily activities and attend his daughter’s upcoming wedding. Although his physical health continued to decline, the psychosocial and spiritual support provided comfort to both the patient and his family during the final months.

After four months from the initial diagnosis, the patient suffered a sudden desaturation and cardiac arrest, leading to his passing. The family, having been prepared through prior counseling, accepted his peaceful death with a sense of closure.

## Discussion

This case of metastatic RCC highlights the importance of palliative care in addressing complex physical, psychological, and emotional needs. While the focus in RCC is often disease management, this case demonstrates the significance of symptom control, psychosocial support, and quality of life, especially in advanced stages where curative options are limited.

The patient’s breathlessness, one of the most distressing symptoms, was managed with tablet morphine (2.5 mg Q4H and PRN), nebulization with Duolin and Budecort, and chest physiotherapy. A pigtail insertion was performed after CECT revealed pleural effusion, offering additional symptomatic relief. Morphine not only helped alleviate dyspnea but also addressed his generalized pain, showcasing the role of opioids in palliative care. As kidney function deteriorated (serum creatinine: 1.20 mg/dL), his pain regimen was adjusted from oral morphine to a fentanyl patch (25 mcg/hr Q72H) for continuous pain control, with sublingual buprenorphine 0.2 mg for BTP. This shift ensured that his pain was well-managed with minimal adverse effects.

Throughout his course of treatment, symptom management remained the primary focus of care. Nausea and vomiting, common side effects, were addressed with the injection of Perinorm 10 mg IV TDS, while syrup laxatives (a stimulant-lubricant combination) were used to manage opioid-induced constipation. The palliative care team continually reassessed his symptoms and adjusted medications to meet his changing needs, ensuring a balance between symptom relief and comfort.

Sudden onset delirium, which can be distressing for both patients and caregivers, was treated effectively with the injection of Haloperidol (0.25 mg HS for eight days). This intervention allowed the patient to regain cognitive clarity, reducing agitation and distress. Lymphedema, caused by hypoproteinemia, was managed with daily magnesium sulfate and glycerine dressings, which reduced swelling by creating an osmotic gradient. This non-invasive approach provided significant comfort and improved mobility. Magnesium also reduced inflammation, improved nerve and muscle function, and prevented arterial hardening, while sulfates facilitated nutrient absorption and detoxification [12].

As part of the palliative care approach, occupational therapy was recommended to help the patient with fatigue, lower limb weakness, and myopathy. Multivitamins, thiocolchicoside, and dexamethasone were prescribed to alleviate weakness and fatigue. An MRI brain scan ruled out any neuropathic cause for the myopathy, reassuring both the patient and caregivers. These interventions ensured that, despite his declining condition, the patient could maintain some degree of function and dignity.

The patient’s clinical course was complicated by progressive thrombocytopenia, a known complication in RCC, which persisted despite treatment with high-dose corticosteroids (dexamethasone 40 mg for four days), romiplostim (1-3 μg/kg), SDP transfusions, and cessation of Sunitinib. The lack of response to these treatments ruled out immune thrombocytopenia (ITP) or Sunitinib-induced thrombocytopenia. Unfortunately, the thrombocytopenia could not be reversed, and the patient experienced sudden desaturation and cardiac arrest, passing away four months after the initial diagnosis.

The palliative care team provided comprehensive psychosocial support. MSW counseled both the patient and his son, helping them understand the intent of care and the prognosis. The son was actively involved in decision-making, ensuring that treatment aligned with the patient’s values. Financial concerns were addressed through family support and the CGHS, allowing the patient to focus on his comfort and well-being. However, one unfulfilled emotional need was the patient’s desire to attend his daughter’s wedding, which took place a week before his death. Despite the best efforts of the care team, this unaccomplished goal remained a poignant reminder of the emotional impact of terminal illness.

The holistic approach of the palliative care team ensured that the end-of-life journey was peaceful and dignified. The family was prepared for the eventual outcome, and they expressed acceptance of his death. This underscores the value of early palliative care integration, ensuring that patients and families are supported throughout the course of illness and can achieve closure when death becomes imminent.

This case also highlights the challenges of managing RCC-associated thrombocytopenia, where conventional treatments, such as steroids and platelet transfusions, were ineffective. For non-candidates of nephrectomy, palliative care becomes even more essential to focus on comfort, rather than cure. While Sunitinib is often associated with thrombocytopenia, discontinuation of the drug in this patient did not reverse the condition, pointing to the limitations of current treatment options.

First-line therapy for RCC-associated ITP typically focuses on the underlying malignancy. Complete responses have been achieved with nephrectomy, with or without concurrent splenectomy, although preoperative treatment with steroids and/or IVIG is often necessary in some cases [13,14]. In patients with metastatic RCC, steroids are usually considered the best treatment option [14], as seen in this case. Despite using steroids and other treatments, the patient’s platelet counts continued to decline, highlighting the limited efficacy of current therapies.

With further depletion in platelet counts, the patient was treated with SDP and romiplostim, yet severe thrombocytopenia persisted until his passing. This underscores the need for better treatment approaches for RCC-associated thrombocytopenia, especially in patients where nephrectomy is not an option.

Thus, this case emphasizes the critical role of palliative care in managing both physical and emotional suffering in patients with advanced cancer. The focus on symptom relief, psychosocial support, and dignity in death underscores the importance of early palliative care involvement, particularly in patients with poor prognosis indicators such as persistent thrombocytopenia, IMDC scores >3/5, pleural effusion, and uncorrected calcium levels [15]. Effective palliative care is not just about extending life but ensuring that patients live as comfortably and meaningfully as possible until the end. Moreover, Sunitinib-induced thrombocytopenia, although well-recognized, demands closer attention, as chemotherapy-related thrombocytopenia remains a significant challenge in RCC care [16].

This case serves as a reminder of the complex interplay between disease-directed treatment and palliative care, urging clinicians to shift the focus toward quality of life when curative options are no longer feasible.

## Conclusions

This case underscores the essential role of palliative care in managing complex symptoms and improving the quality of life for patients with advanced RCC. It highlights the need for a holistic approach that integrates symptom relief, psychosocial support, and caregiver education throughout the patient’s journey. Despite aggressive treatments for associated complications like thrombocytopenia, the patient's persistent symptoms demonstrated the limitations of curative intent in the context of advanced disease. Through comprehensive palliative care, the patient's physical, emotional, and spiritual needs were prioritized, enabling a dignified end-of-life experience. The involvement of a multidisciplinary team, including medical social workers and palliative care specialists, was crucial in addressing not only the medical complexities but also the emotional and financial concerns of the patient and family.

As the field of oncology evolves, the integration of palliative care from the time of diagnosis remains imperative, especially for patients with poor prognostic indicators. This case serves as a reminder of the importance of focusing on patient-centered care, ensuring that individuals can navigate their illness with dignity, comfort, and support, even when faced with the inevitability of death. Future efforts should aim to explore and establish more effective treatment protocols for managing complications like thrombocytopenia in patients with metastatic RCC while continuing to prioritize their quality of life.
